# Detecting short-interval longitudinal cortical atrophy in neurodegenerative dementias via cluster scanning: A proof of concept

**DOI:** 10.1162/IMAG.a.1138

**Published:** 2026-02-20

**Authors:** Yuta Katsumi, Michael Brickhouse, Lindsay C. Hanford, Jared A. Nielsen, Maxwell L. Elliott, Ross W. Mair, Alexandra Touroutoglou, Mark C. Eldaief, Randy L. Buckner, Bradford C. Dickerson

**Affiliations:** Frontotemporal Disorders Unit, Department of Neurology, Massachusetts General Hospital and Harvard Medical School, Boston, MA, United States; Department of Psychology, Center for Brain Science, Harvard University, Cambridge, MA, United States; Department of Psychology, Neuroscience Center, Brigham Young University, Provo, UT, United States; Athinoula A. Martinos Center for Biomedical Imaging, Massachusetts General Hospital, Charlestown, MA, United States; Massachusetts Alzheimer’s Disease Research Center, Massachusetts General Hospital and Harvard Medical School, Charlestown, MA, United States; Department of Psychiatry, Massachusetts General Hospital and Harvard Medical School, Boston, MA, United States

**Keywords:** Alzheimer’s disease, frontotemporal lobar degeneration, magnetic resonance imaging, precision neuroimaging, cortical thickness

## Abstract

Regional brain atrophy estimated from structural magnetic resonance imaging (MRI) is a widely used measure of neurodegeneration in Alzheimer’s disease (AD), Frontotemporal Lobar Degeneration (FTLD), and other dementias. Yet, traditional MRI-derived morphometric estimates are susceptible to measurement errors, posing a challenge for detecting longitudinal atrophy over short intervals. Here, we examined the utility of multiple MRI scans acquired in rapid succession (i.e., *cluster scanning*) for detecting longitudinal cortical atrophy over 3- and 6-month intervals within individual participants. Four individuals with mild cognitive impairment or mild dementia likely due to AD or FTLD participated in this study. At baseline, 3 months, and 6 months, structural MRI data were collected on a 3 Tesla scanner using a fast 1.2-mm T1-weighted multi-echo magnetization-prepared rapid gradient echo (MEMPRAGE) sequence (acquisition time = 2’23”). At each timepoint, participants underwent up to 32 MEMPRAGE scans acquired in four separate sessions over 2 days. Using linear mixed-effects models, we found that phenotypically vulnerable cortical (“core atrophy”) regions exhibited statistically significant longitudinal atrophy in all participants (i.e., decreased cortical thickness) by 3 months and further demonstrated preferential vulnerability compared to control regions in three of the participants over at least one of the 3-month intervals. These findings provide proof-of-concept evidence that pooling multiple morphometric estimates derived from cluster scanning can detect longitudinal cortical atrophy over short intervals in individual patients with neurodegenerative dementias.

## 1 Introduction

Regional brain atrophy estimated from structural magnetic resonance imaging (MRI) is a widely used measure of neurodegeneration in Alzheimer’s disease (AD), Frontotemporal Lobar Degeneration (FTLD), and other dementias. There is now ample evidence to suggest that MRI-derived brain atrophy estimates are useful for diagnosis, prognostication, and longitudinal outcome monitoring in neurodegenerative disease. For example, we and others previously identified spatially distinct “signature” patterns of cortical atrophy in early symptomatic patients with late-onset AD (Dickerson et al., 2009; Jack & Holtzman, 2013; Jack et al., 2015), early-onset AD (Frisoni et al., 2007; Harper et al., 2017; Katsumi et al., 2025; Möller et al., 2013; Touroutoglou et al., 2023), and various clinical phenotypes of FTLD (Agosta et al., 2015; Caso et al., 2014; Eldaief et al., 2023; Harper et al., 2017; Rohrer et al., 2011; Seeley et al., 2008; Vemuri et al., 2011; Zhang et al., 2013), which are highly replicable across independent samples and robustly associated with symptom severity.

Regional atrophy patterns are less robust and consistent in Dementia with Lewy bodies (Harper et al., 2017; Vemuri et al., 2011; Ye et al., 2020). The magnitude of MRI-based cortical atrophy in these signature regions and other phenotypically vulnerable brain networks can predict subsequent clinical progression or decline among individuals at early clinical stages of AD or FTLD (Anderl-Straub et al., 2021; Bakkour et al., 2009; Dickerson & Wolk, 2013; Katsumi et al., 2023, 2024; Keret et al., 2021; Staffaroni et al., 2019) as well as individuals who develop these syndromes but were scanned when cognitively unimpaired (Dickerson et al., 2011; Prosser et al., 2023).

Longitudinal MRI studies have demonstrated unique spatiotemporal characteristics of brain atrophy progression across phenotypic subtypes of AD and FTLD, while also identifying the relationship between the pattern of longitudinal atrophy and other imaging biomarkers of neuropathologic change (Bejanin et al., 2020; Harrison et al., 2019; La Joie et al., 2020; Phillips et al., 2019; Ranasinghe et al., 2021; Sintini et al., 2019, 2020). In addition to its utility for morphometric analyses, structural MRI is commonly used as an anatomical reference for other neuroimaging modalities such as positron emission tomography (PET), functional MRI, or diffusion MRI data, further highlighting the importance of obtaining reliable morphometrics for the accurate characterization of each patient’s brain structural integrity.

Traditional MRI measures of brain atrophy can reveal submillimeter structural abnormalities in neurodegenerative patients. However, MRI-derived morphometric estimates are prone to small-magnitude measurement errors, which pose a significant challenge for reliably detecting longitudinal brain structural change within an individual patient, particularly over short time intervals. Common morphometric estimates (e.g., regional cortical thickness generated by FreeSurfer) in healthy participants have measurement errors of 2–5%, calculated based on repeat scans acquired during the same imaging session (Tustison et al., 2014) or in sessions a few weeks apart (Dickerson et al., 2008; Han et al., 2006; Jovicich et al., 2009; Wonderlick et al., 2009). The annual rate of atrophy in the hippocampus and late-onset AD signature cortical regions is ~2–6% in symptomatic AD patients (Barnes et al., 2009; Fjell et al., 2009; Schuff et al., 2009). Therefore, the magnitude of longitudinal atrophy over 1 year is comparable on average to that of measurement errors.

**Fig. 1.**
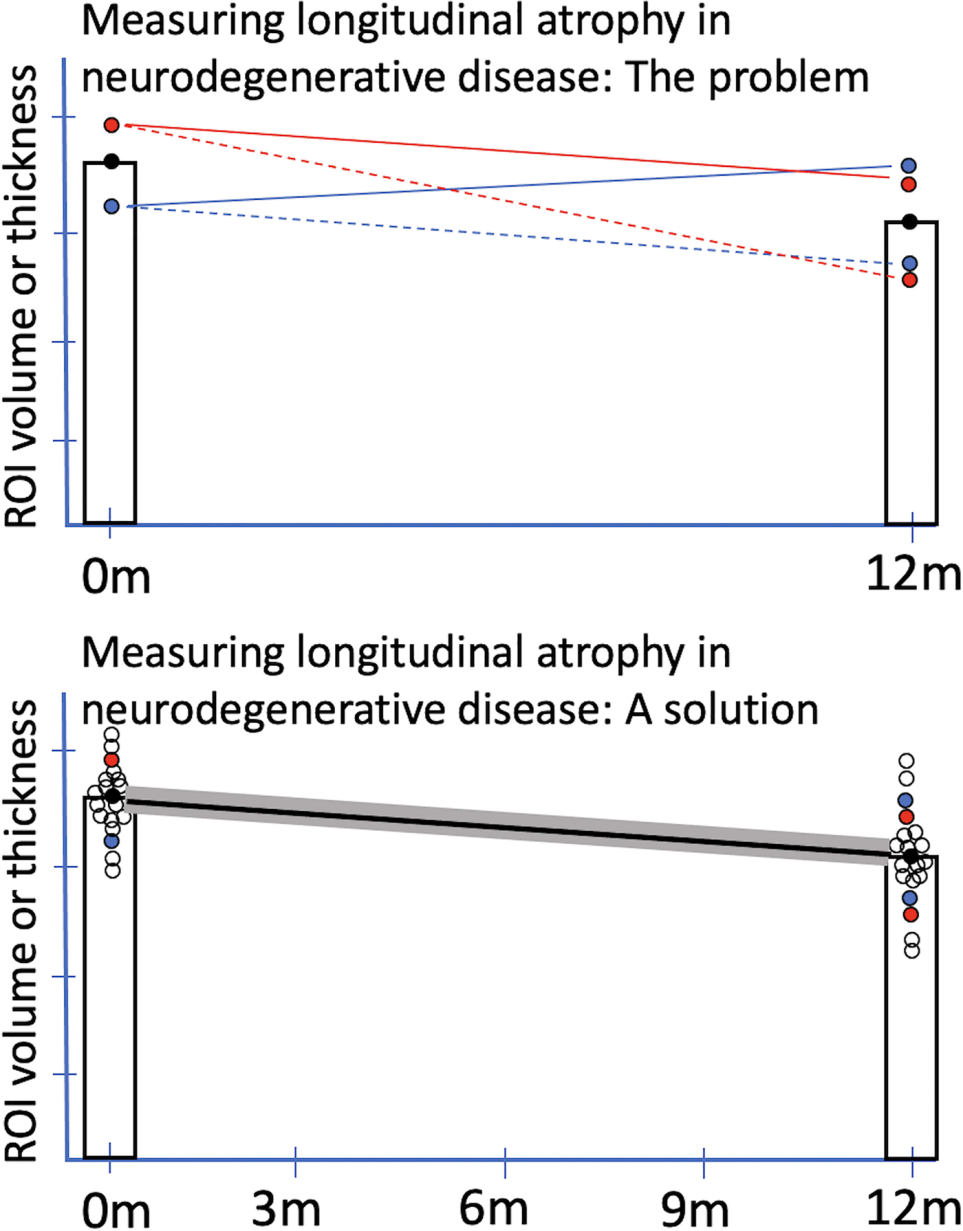
Detection of longitudinal atrophy in neurodegenerative disease. Estimates of longitudinal atrophy rates in neurodegenerative disease are imprecise with current methods, primarily because of undersampling. The top graph illustrates the problem. At each timepoint, a single T1-weighted (T1w) structural MRI scan is typically acquired. It is clear from a variety of studies, including our own, that various sources of error contribute to a distribution of values around the central tendency (represented by black dot). If the baseline estimate is high (red dot), the annualized atrophy rate may be estimated reasonably well at 12-month follow-up (solid red line) or overestimated (dashed red line). If the baseline estimate is low (blue dot), the atrophy rate may be estimated reasonably well (dashed blue line) or may be grossly underestimated (solid blue line). The bottom graph illustrates a proposed solution based on cluster scanning (Elliott et al., 2025). Dense sampling via cluster scanning at each timepoint should enable not only more precise estimates at typical intervals for longitudinal scanning, but also potentially more sensitive detection of atrophy at shorter intervals, including 6-month or even 3-month intervals.

Measuring brain atrophy over short time intervals (<1 year) has important implications for clinical trial design, as it would allow neurodegeneration to be monitored in smaller, early-phase therapeutic trials, particularly those targeting patients with relatively rapidly progressive forms of dementia (e.g., early-onset AD, behavioral variant FTD) (Lima-Silva et al., 2021; Mendez, 2019). However, the feasibility of reliably estimating short-term atrophy is limited using conventional MRI scans, as most contemporary longitudinal studies of neurodegenerative dementias collect only one or two of these scans annually, such as the Alzheimer’s Disease Neuroimaging Initiative (ADNI) (Veitch et al., 2023), the Dominantly Inherited Alzheimer Network (DIAN) (Moulder et al., 2013), the ARTFL LEFFTDS Longitudinal Frontotemporal Lobar Degeneration study (ALLFTD) (Boeve et al., 2020), the Longitudinal Early-onset Alzheimer’s Disease Study (LEADS) (Apostolova et al., 2021), and the Parkinson Progression Marker Initiative (PPMI) (Marek et al., 2011). Point estimates based on single MRI acquisitions are subject to normal variation around the central tendency, which may lead to over- or underestimation of atrophy rates over time in any given individual (Fig. 1).

To improve brain morphometric precision, we previously introduced the concept of *cluster scanning*, which involves pooling estimates by taking the average of multiple repeated measurements acquired from an individual at essentially one timepoint (Elliott et al., 2024, 2025; Nielsen et al., 2019). While still uncommon in structural neuroimaging, achieving greater measurement precision through repeated sampling and data aggregation is an established approach in functional neuroimaging (Birn et al., 2013; Elliott et al., 2019; Gordon et al., 2017; Laumann et al., 2015), psychophysiology (Boudewyn et al., 2018; Huffmeijer et al., 2014), and psychometrics (Crocker & Algina, 1986; Kuder & Richardson, 1937; Nicosia et al., 2023). In a recent study employing the cluster scanning protocol, we compared brain morphometrics estimated from a single standard T1w magnetization-prepared rapid gradient echo (MPRAGE) scan using the ADNI sequence to pooled estimates from multiple accelerated MRI scans collected in succession from a mixed sample of healthy young and older participants as well as symptomatic patients with MCI or dementia likely due to AD or FTLD. We found that pooling estimates from four accelerated MRI scans yields an average 34% reduction in measurement error compared with estimates based on a single ADNI scan, where the error was defined as the absolute difference between each morphometric measure estimated from two separate sessions on separate days divided by the mean of the two measurements (Elliott et al., 2024). Comparable benefits from pooling estimates were identified for both subcortical volumetric and cortical thickness measures. Importantly, the magnitude of error reductions by pooling estimates was comparable across healthy young adults (33%), healthy older adults (34%), and AD/FTLD patients (35%). Taken together, these findings show that cluster scanning improves morphometric precision by reducing measurement error and its utility is clearly demonstrated in patient populations where poorer data quality may be expected.

In this pilot study, we performed a proof-of-concept investigation of the utility of cluster scanning to detect short-interval longitudinal cortical atrophy within individual patients diagnosed with MCI or mild dementia likely due to AD or FTLD. Augmenting our prior work (Elliott et al., 2024, 2025), we focus here on identifying atrophy patterns in the baseline scans and using them to understand the idiosyncratic atrophy patterns that evolve within the brains of individual patients over time. At the time of study execution, some of the accelerated MRI sequences above were not yet readily available. Therefore, each patient (*N* = 4) underwent repeated fast low-resolution (1.2 mm) multi-echo MPRAGE (MEMPRAGE) acquisitions (Holmes et al., 2015; Mair et al., 2012) across three time points over a 6-month period. We then calculated the rate of longitudinal atrophy in each patient over 3- and 6-month intervals based on pooled morphometric estimates obtained from up to 32 scans per time point. We hypothesized that longitudinal cortical atrophy would be detectable over 3- and 6-month intervals within individual patients with AD or FTLD, thus demonstrating the utility of cluster scanning for detecting longitudinal short-interval atrophy. Furthermore, we hypothesized that the magnitude of longitudinal cortical atrophy would be greater in phenotypically more vulnerable (i.e., core atrophy) regions than in less vulnerable regions uniquely defined in each patient with AD or FTLD, thus demonstrating the utility of cluster scanning for detecting selective longitudinal short-interval atrophy in core regions.

## 2 Methods

### 2.1 Participants

Four participants with MCI or mild dementia (Clinical Dementia Rating, CDR = 0.5 or 1) likely due to AD (*n* = 3; with positive amyloid, tau, and neurodegeneration imaging biomarkers) (Jack et al., 2016) or FTLD (*n* = 1) were recruited through the Massachusetts General Hospital Frontotemporal Disorders Unit. Of the three AD patients, two of them clinically presented with logopenic variant Primary Progressive Aphasia (lvPPA) (Gorno-Tempini et al., 2011), while one presented with the Posterior Cortical Atrophy (PCA) syndrome (Crutch et al., 2017; Mendez et al., 2002; Tang-Wai et al., 2004). The FTLD patient’s presentation was semantic variant PPA (svPPA) (Gorno-Tempini et al., 2011). We chose this group of participants to explore the viability of longitudinal cluster scanning across individuals with distinct patterns and etiologies of atrophy (Gorno-Tempini et al., 2004; Mummery et al., 2000; Whitwell et al., 2007). CDR global, CDR Sum-of-Boxes (CDR-SB), and FTLD-adapted CDR-SB (FTLD-CDR-SB) scores (Knopman et al., 2008) were obtained from recent clinical or research visits. See Table 1 for demographic and clinical characteristics of the sample. The participants self-reported that their ethnicity and race were non-Hispanic Caucasian.

**Table 1.** Demographic and clinical characteristics of the sample.

Participant ID	lvPPA-1	lvPPA-2	PCA	svPPA
Likely etiology	AD	AD	AD*	FTLD**
Sex	M	M	F	M
Age at baseline	69.5	74.0	59.7	70.8
Years of education	20	20	16	18
Years since symptom onset	5	8	6	7
CDR global	1	0.5	0.5	0.5
CDR-SB	4	3	3	0.5
FTLD-CDR-SB	5	4	3	1

* Autopsy-proven AD pathology.

** Autopsy-proven FTLD TDP43 pathology.

Notes: AD = Alzheimer’s Disease; FTLD = Frontotemporal Lobar Degeneration; CDR = Clinical Dementia Rating; CDR-SB = Clinical Dementia Rating Sum-of-Boxes; lvPPA = logopenic variant Primary Progressive Aphasia; svPPA = semantic variant Primary Progressive Aphasia; PCA = Posterior Cortical Atrophy.

### 2.2 MRI data acquisition

MRI data used to assess longitudinal atrophy in each participant were collected at the Harvard Center for Brain Science using a 3T Siemens MAGNETOM Prisma^fit^ MRI scanner (Siemens Healthineers AG; Erlangen, Germany) and the vendor’s 64-channel head coil. Specifically, we utilized a fast T1w MEMPRAGE sequence (henceforth *fast* structural MRI): Acquisition time = 2’23”, repetition time [TR] = 2.2 seconds, echo times [TEs] = 1.57/3.39/5.21/7.03 ms, flip angle = 7°, inversion time [TI] = 1.1 seconds, slice orientation = sagittal, matrix size = 192 × 192 × 144, in-plane generalized auto-calibrating partial parallel acquisition (GRAPPA) acceleration factor = 4, and voxel size = 1.2 mm isotropic voxels (Holmes et al., 2015; Mair et al., 2012). This sequence has been shown to yield morphometric estimates that are highly consistent (*R*^2^ > 0.9 for several gray and white matter structures) with those obtained from more conventional MPRAGE scans with longer acquisition time and higher spatial resolution (Mair et al., 2012). All fast structural MRI scans were acquired with motion-tracking enabled via volumetric navigators (vNavs) (Reuter et al., 2015; Tisdall et al., 2016) to provide estimates of head motion in each scan, although prospective motion correction was not performed. During the scanning sessions, participants were encouraged to remain still and given the option to watch video clips (e.g., a nature documentary) or to listen to music. Inflatable cushions were used to provide additional hearing protection and to immobilize the participants’ heads. Every 5–10 minutes, participants were given feedback about motion and reminded to stay still.

The cluster scanning protocol collected up to 32 fast structural MRI scans per participant across three timepoints (baseline, 3-month, and 6-month follow-up). At each timepoint, participants completed four scanning sessions over 2 days (mean interval = 4.5 ± 1.88 days). Each day included two sessions: In the first session, we collected eight fast structural MRI scans plus additional structural and functional acquisitions (not analyzed here). After a brief break for stretching and restroom use, participants were repositioned for a second session where we repeated the same protocol with another eight fast structural MRI scans. This yielded 16 scans per day, or 32 scans per timepoint, with each visit lasting no more than 2 hours total. Due to technical difficulties, we lost half of the scans acquired from the PCA participant at baseline.

We additionally acquired from each participant a *standard* structural MRI scan via a conventional MEMPRAGE sequence at the MGH Athinoula A. Martinos Center for Biomedical Imaging using a 3T Siemens Tim Trio MRI scanner and the vendor’s 12-channel head coil: Acquisition time = 5’53”, TR = 2.53 seconds, TEs = 1.64/3.5/5.36/7.22 ms, flip angle = 7°, TI = 1.2 seconds, slice orientation = sagittal, matrix size = 256 × 256 × 176, GRAPPA acceleration factor = 2, and voxel size = 1 mm isotropic voxels. These data were collected as part of their participation in different studies within 1 year preceding the baseline of the present study (mean time before baseline = 7.7 ± 4.7 months) and were used to independently define regions of interest (ROI) masks for the analysis of longitudinal cortical atrophy over 3- and 6-month periods (see Section 2.4 below).

### 2.3 MRI data preprocessing

Each participant’s standard structural MRI scan was processed with FreeSurfer version 6.0.0 using the conventional cross-sectional recon-all pipeline, which involved intensity normalization, skull stripping, and an automated segmentation of cerebral white matter to locate the gray matter/white matter boundary. Defects in the surface topology were corrected (Fischl et al., 2001), and the gray/white boundary was deformed outward using an algorithm designed to obtain an explicit representation of the pial surface. We visually inspected each participant’s cortical surface reconstruction for technical accuracy.

To estimate the rates of longitudinal atrophy, each participant’s fast structural MRI scans were processed with FreeSurfer’s longitudinal recon-all pipeline (Reuter et al., 2012). Specifically, fast structural MRI scans from each timepoint were first processed via the cross-sectional recon-all pipeline, after which an unbiased, within-subject template space and image were created using robust, inverse consistent registration using all longitudinal fast MRI scans available to each participant (Reuter et al., 2010; Reuter & Fischl, 2011). Several processing steps, such as skull stripping and atlas registration, as well as spherical surface maps and parcellations were then initialized with common information from the within-subject template (Reuter et al., 2012). For each standard and fast structural MRI scan, cortical thickness was calculated as the average of the distance from the gray/white boundary to the closest point on the gray/cerebrospinal fluid boundary and from that point back to the closest point on the gray/white boundary at each vertex on the tessellated surface (Fischl & Dale, 2000). We resampled each participant’s native-space cortical thickness maps to *fsaverage* space (with ~160 k vertices per hemisphere) and geodesically smoothed these resampled thickness maps with FWHM = 15 mm.

We examined head motion estimates calculated by vNavs for fast structural MRI scans obtained in clusters. During each TR, the vNavs system estimates the participant’s head displacement relative to the first TR of the scan. To summarize head motion, we computed the root mean square (RMS) of the displacement over all points inside a sphere with 64 mm radius, initially centered at isocenter (Jenkinson, 1999), resulting in one summary metric of estimated head motion per TR. The framewise motion estimates were then averaged over the duration of the scan, which yielded a single motion score per scan, RMS displacement per minute (RMSpm). In this study, we discarded from each participant all scans with RMSpm > 10 mm/min. The number of scans discarded based on this criterion for each participant was: lvPPA-1 = 5 (~5%), lvPPA-2 = 1 (~1%), PCA = 13 (~16%), and svPPA = 0 (0%).

### 2.4 ROI mask creation

Given that our participants were in the early clinical stages of AD or FTLD, we hypothesized that they would continue to exhibit longitudinal atrophy in cortical regions where some atrophy had already been present prior to their participation in this study. To identify these phenotypically vulnerable cortical regions tailored to the idiosyncratic anatomy of each participant, we first processed the standard structural MRI scan acquired independently of the cluster scanning data following the procedure described above. We then converted whole-cortex vertex-wise estimates of cortical thickness to *W*-scores (Jack et al., 1997; Katsumi et al., 2023, 2025). *W*-scores are analogous to *Z*-scores adjusted for specific covariates of no interest, which in this study were participants’ age and sex. Separately for each vertex, we first constructed a multiple linear regression model using cortical thickness data obtained from a group of biomarker-confirmed amyloid-negative (Aβ-) control participants (mean age = 67.4 ± 4.8, 13 men/12 women) (Katsumi et al., 2023), with the following formula:

$${\hat{T}}_{ij}=\beta_{0}+\beta_{1}Age+\beta_{2}Sex+\varepsilon$$

where ${\hat{T}}_{ij}$ = the predicted cortical thickness at vertex i and for Aβ- control participant j. This regression model produced beta coefficient values for age and sex and individual values of residuals. Using these parameters, we then computed *W*-scores for each vertex and participant with the following formula:

$$W_{ij}=\;\frac{T_{ij}-\;{\hat{T}}_{ij}}{SD_{i}}$$

where $T_{ij}$ = the observed cortical thickness at vertex i and for participant j, ${\hat{T}}_{ij}$ = the predicted cortical thickness at vertex i and for patient j based on age and sex of the participant and beta coefficients obtained from Aβ- controls, and $SD_{i}$ = the standard deviation of the individual residuals obtained from Aβ- controls for vertex i. Because *W*-scores in this study were calculated using cortical thickness, more negative values indicate greater cortical atrophy relative to what would be expected solely based on age and sex of each participant.

For each participant, we defined two types of ROIs.

#### 2.4.1 Core atrophy ROIs

To define phenotypically vulnerable (“core” atrophy) ROIs, we first identified regions showing relatively greater atrophy by thresholding each participant’s *W*-score atrophy map at *W* < -2. Using FreeSurfer’s tksurfer tools, we visually inspected these thresholded maps and manually selected clusters that anatomically corresponded to regions where prominent neurodegeneration is typically observed in each AD or FTLD clinical syndrome (Crutch et al., 2017; Gorno-Tempini et al., 2004, 2011). For two participants, we used anatomical parcellation labels generated by FreeSurfer (Desikan et al., 2006) to aid in anatomical localization.

#### 2.4.2 Control ROIs

Separately, we defined “control” ROIs as cortical regions expected to show minimal longitudinal atrophy. We began with the precentral and postcentral gyri (primary motor and somatosensory cortex) from each participant’s FreeSurfer cortical parcellation, as these regions are typically spared in lvPPA, svPPA, and PCA. To ensure that these control ROIs, indeed, contained minimal atrophy, we removed any vertices with *W* < -0.25 from the precentral/postcentral labels.

**Fig. 2.**
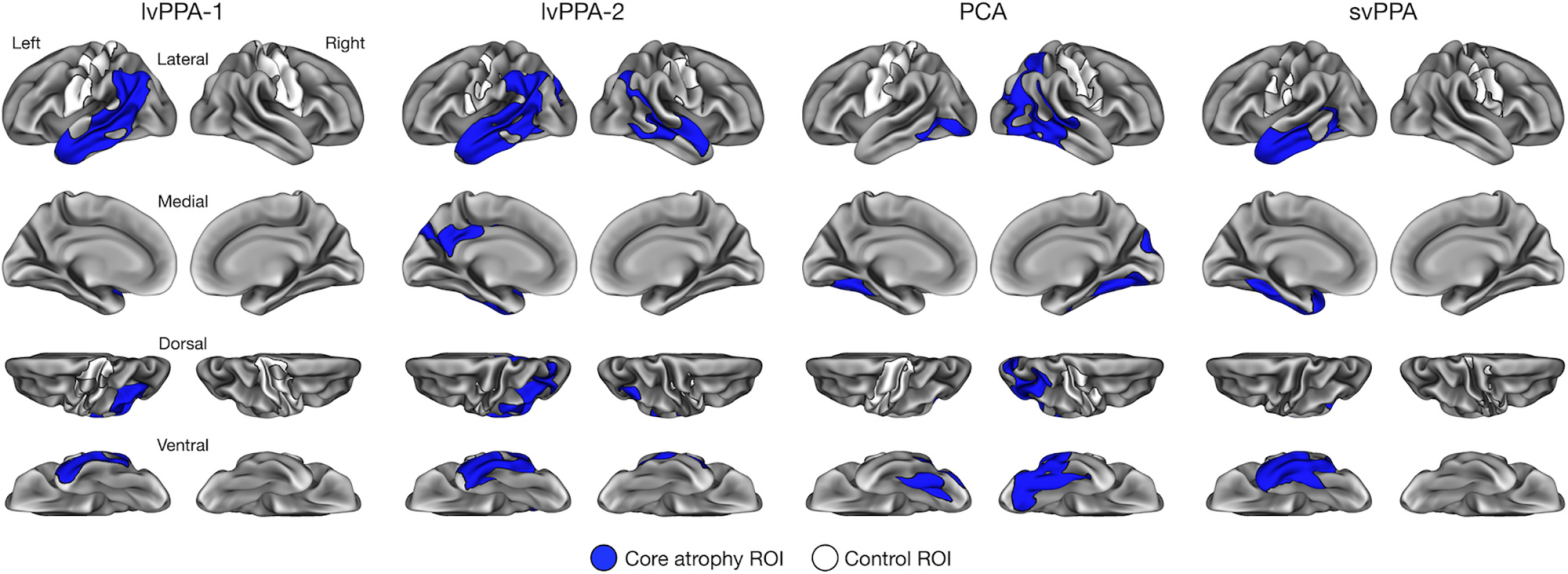
Individualized cortical thinning signatures (iCORTS) ROI masks. For each participant, core atrophy ROI (blue) and control ROI (white) masks were defined based on a standard structural MRI scan collected prior to their participation in the current study (see Section 2.4).

The final ROI masks used for statistical analysis of each participant’s fast structural MRI data are shown in Figure 2. For each fast structural MRI scan, mean cortical thickness of each ROI was calculated by averaging thickness values at all vertices falling within its boundaries.

### 2.5 Statistical analysis

To examine longitudinal cortical atrophy, we constructed linear mixed-effects models separately for each individual participant using the *lme* function from the *nlme* package (version 3.1-164) (Pinheiro et al., 2023) run on *R* version 4.4.2. These models were fit by maximizing the restricted likelihood and included Timepoint (baseline, 3 months, and 6 months) and ROI (core and control) as fixed predictors of interest, estimated head motion (RMSpm) as a covariate of no interest, and nested random intercepts for scans within session within day. For each participant, we additionally fitted a simpler model with random intercepts for scans only (i.e., no explicit modeling of session and day effects) and conducted likelihood ratio tests to compare model performance. In the case of negligible improvement in model fit (*p* > .05), we retained the simpler model for subsequent analyses. Furthermore, we included variance weights in these models to allow for heteroscedasticity in cortical thickness estimates between core atrophy and control ROIs across timepoints.

To test the hypothesis about the utility of cluster scanning for short-interval atrophy detection, we examined the main effect of Timepoint estimated by each model. Planned comparisons investigated pairwise differences in cortical thickness separately for core atrophy and control ROIs using the *emmeans* package (version 1.10.0). We then examined the interaction effect between Timepoint and ROI to test the hypothesis that phenotypically vulnerable regions would exhibit greater atrophy than control regions. If a significant interaction was identified, we computed the magnitude of longitudinal atrophy between a given pair of timepoints separately for core atrophy and control ROIs and compared these estimates. *P*-values were Bonferroni-corrected for multiple statistical comparisons within each participant based on the number of contrasts in question.

Additionally, we performed an exploratory vertex-wise analysis of longitudinal cortical atrophy within the boundaries of each participant’s core atrophy ROIs. This analysis allowed us to examine potential subregional specificity in the localization of longitudinal atrophy and how it might change over different time intervals. Using vertex-wise cortical thickness data as inputs, we constructed linear mixed-effects models separately for each vertex within each participant’s core atrophy ROIs. Given the number of vertices involved in this analysis, it is possible that the nested random-effects structure might be too complex for some of them, resulting in singular fit. To overcome this potential issue, we initialized model estimation for all vertices with the nested random intercepts for scans within session within day as described above. In the case of singular fit, the model was refit by only retaining random intercepts for scans. If this model also resulted in singular fit, a fixed-effects-only model was fit to the data. Using vertex-wise estimated marginal means, we calculated surface maps representing the percent change in cortical thickness from one timepoint to another for the three pairwise contrasts: Baseline versus 3 months, 3 months versus 6 months, and Baseline versus 6 months. The magnitude of change was considered statistically significant at each vertex if *p* < .05 for the main effect of Timepoint and *p* < .017 for the corresponding pairwise contrast (Bonferroni-corrected for the number of comparisons). Due to the exploratory nature of this analysis, we did not otherwise adjust *p*-values for the number of vertices within each participant’s ROIs.

Finally, to assess the relationship between scan quantity and atrophy estimation precision, we examined how the number of fast structural MRI scans per timepoint influences the precision of cortical atrophy estimated across random data samples. In this analysis, we focused on longitudinal atrophy estimates from baseline to 3- and 6-month follow-ups, where significant atrophy was observed in core ROIs across all participants. For each participant and interval, we first established the reference atrophy rate by averaging 10,000 bootstrap estimates of thickness difference, where each bootstrap sample was created by randomly sampling 32 scans with replacement per timepoint. The resulting reference atrophy rate represented our best estimate of the true population parameter given the available data. We then quantified estimation precision across numbers of scans (1–32 per timepoint) by calculating the mean percent deviation between bootstrap atrophy estimates and the reference atrophy rate. Specifically, percent deviation was defined as the absolute difference between each bootstrap estimate (at a given number of scans) and the reference atrophy rate, divided by the reference rate, with larger values indicating greater deviation from the reference atrophy rate and therefore lower precision. Based on this metric, we then estimated the number of fast structural MRI scans at which point the reduction in percent deviation was minimal. This was accomplished by identifying the point of sustained diminishing returns along the percent deviation curve, where the reduction in percent deviation was less than 5% of the immediately preceding deviation value for at least two consecutive steps. That is, a minimum of 10 scans would mean that both the deviation reduction from 8 to 9 and from 9 to 10 fast structural MRI scans would each be no more than 5% of the preceding deviation value for a given participant and interval.

## 3 Results

### 3.1 Cluster scanning detects longitudinal cortical atrophy over 3- and 6-month intervals in individual patients with AD or FTLD

Parameter estimates and statistical test results obtained from linear mixed-effects models are summarized in tables below, including cortical thickness for each ROI, timepoint, and participant (Table 2), results of fixed effects statistical tests (Table 3), and change in cortical thickness across each time interval of interest, separately for core and control ROIs (Table 4).

**Table 2.** Estimated marginal means for cortical thickness by participant, region of interest, and timepoint.

		Timepoint		
Participant ID	ROI	BL	3 months	6 months
lvPPA-1	Core atrophy	1.81 (1.72–1.90)	1.78 (1.69–1.87)	1.76 (1.67–1.85)
	Control	2.15 (2.05–2.25)	2.15 (2.04–2.25)	2.15 (2.05–2.25)
lvPPA-2	Core atrophy	1.93 (1.92–1.94)	1.90 (1.89–1.91)	1.89 (1.88–1.90)
	Control	2.15 (2.13–2.16)	2.11 (0.20–2.13)	2.13 (2.11–2.14)
PCA	Core atrophy	1.89 (1.88–1.90)	1.85 (1.85–1.86)	1.83 (1.83–1.84)
	Control	2.26 (2.24–2.27)	2.26 (2.25–2.28)	2.23 (2.21–2.24)
svPPA	Core atrophy	1.78 (1.77–1.78)	1.76 (1.75–1.76)	1.73 (1.73–1.74)
	Control	2.35 (2.34–2.36)	2.35 (2.34–2.36)	2.34 (2.33–2.35)

Notes: Values indicate estimated marginal means (95% confidence intervals).

**Table 3.** Results of fixed effects statistical tests from linear mixed-effects models.

Participant ID	Fixed effects	*df* (num)	*df* (den)	*F*	*p*
lvPPA-1	Timepoint	2	87	53.13	**<.0001**
	ROI	1	88	26552.89	**<.0001**
	Motion	1	87	2.94	.09
	Timepoint × ROI	2	88	33.25	**<.0001**
lvPPA-2	Timepoint	2	91	33.78	**<.0001**
	ROI	1	92	3711.12	**<.0001**
	Motion	1	91	5.74	**.019**
	Timepoint × ROI	2	92	4.21	**.018**
PCA	Timepoint	2	63	48.42	**<.0001**
	ROI	1	64	12033.93	**<.0001**
	Motion	1	63	2.39	.13
	Timepoint × ROI	2	64	9.14	**<.0001**
svPPA	Timepoint	2	92	45.06	**<.0001**
	ROI	1	93	66375.26	**<.0001**
	Motion	1	92	0.08	.78
	Timepoint × ROI	2	93	15.77	**<.0001**

Note: Bolded values indicate statistically significant effect at *p* < .05.

**Table 4.** Estimated rates of longitudinal change in cortical thickness.

		Time interval		
Participant ID	ROI	from BL to 3 months	from BL to 6 months	from 3 to 6 months
lvPPA-1	Core atrophy	0.029 (0.020 to 0.038) [-1.6%]	0.050 (0.041 to 0.058) [-2.8%]	0.021 (0.013 to 0.029) [-1.2%]
	Control	0.008 (-0.006 to 0.022) [-0.4%]	0.005 (-0.009 to 0.019) [-0.2%]	-0.003 (-0.017 to 0.011) [+0.1%]
lvPPA-2	Core atrophy	0.029 (0.016 to 0.041) [-1.5%]	0.039 (0.028 to 0.05) [-2.0%]	0.01 (-0.001 to 0.021) [-0.5%]
	Control	0.033 (0.012 to 0.055) [-1.5%]	0.019 (-0.002 to 0.04) [-0.9%]	-0.014 (-0.035 to 0.007) [+0.7%]
PCA	Core atrophy	0.032 (0.021 to 0.043) [-1.7%]	0.054 (0.043 to 0.065) [-2.9%]	0.022 (0.013 to 0.032) [-1.2%]
	Control	-0.009 (-0.031 to 0.013) [+0.4%]	0.028 (0.005 to 0.051) [-1.2%]	0.037 (0.018 to 0.056) [-1.6%]
svPPA	Core atrophy	0.020 (0.011 to 0.029) [-1.1%]	0.041 (0.032 to 0.05) [-2.3%]	0.021 (0.012 to 0.03) [-1.2%]
	Control	0.0001 (-0.014 to 0.014) [<-0.1%]	0.01 (-0.005 to0.024) [-0.4%]	0.01 (-0.005 to 0.024) [-0.4%]

Notes: Values indicate estimated magnitudes of change in cortical thickness (95% confidence intervals). Values shown inside square brackets represent the same estimates but expressed as percent change relative to the reference timepoint for a given time interval (BL or 3 months) (see Table 2).

**Fig. 3.**
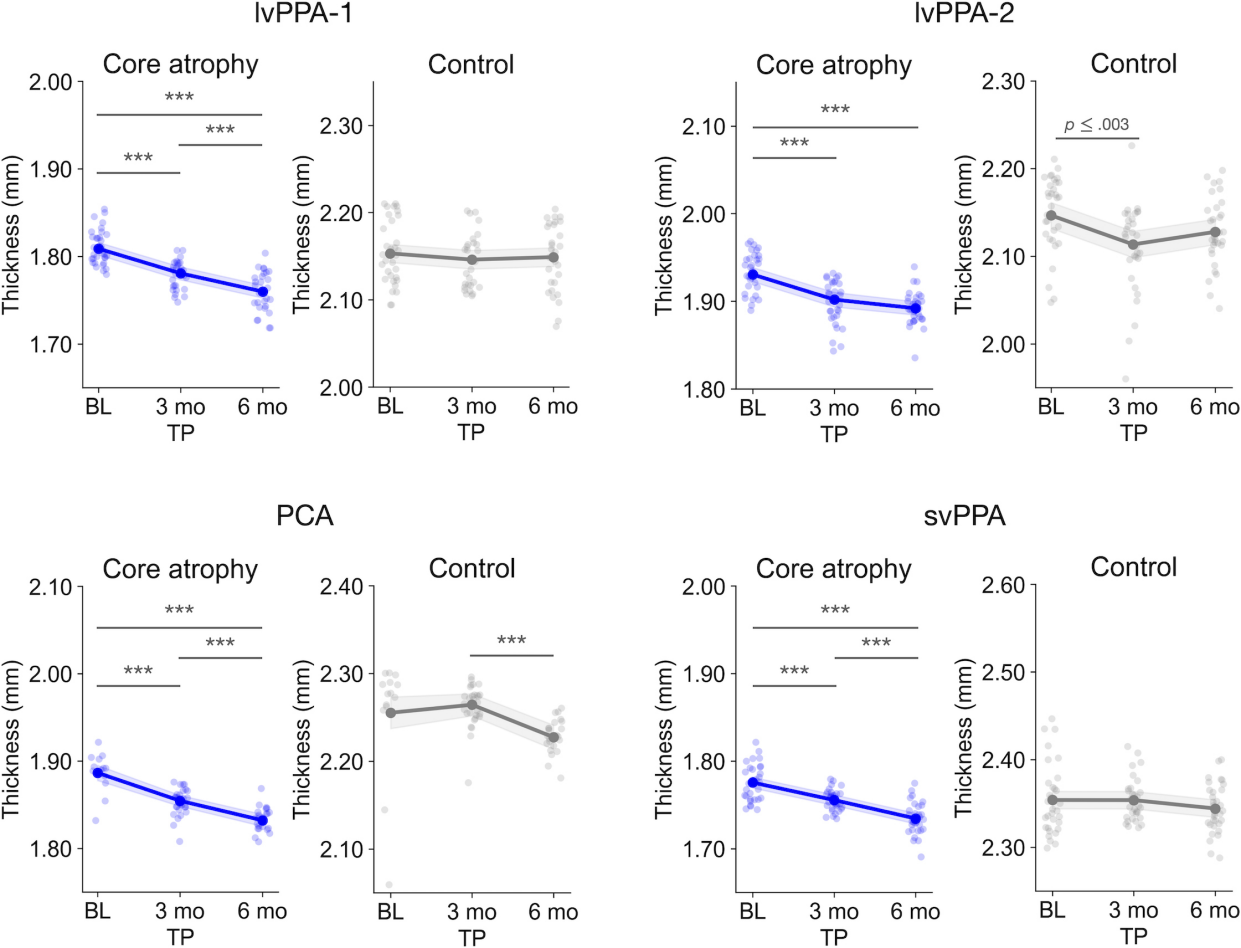
Pooling morphometric estimates via cluster scanning reveals short-interval longitudinal cortical atrophy in neurodegenerative patients. Each plot displays estimated marginal means for cortical thickness at each time point (larger opaque points) obtained from linear mixed-effects models, overlaid on top of raw thickness values derived from individual fast structural MRI scans (smaller transparent points). Shaded bands represent 95% confidence intervals. Statistical significance is denoted only if a given comparison survives a Bonferroni-corrected threshold of *p* < .008 (*p*_bonf_ = .05/6). ***p < .0001. TP = Timepoint; BL = baseline; 3 mo = 3 months; 6 mo = 6 months.

In all four participants, we found a significant main effect of Timepoint (all *p*’s < .0001; see Table 3 for complete statistics). Planned comparisons revealed that, in all participants, core regions showed longitudinal atrophy from baseline to 3 months (lvPPA-1: *t*(84) = 6.54, *p* < .0001; lvPPA-2: *t*(91) = 4.67, *p* < .0001; PCA: *t*(63) = 5.90, *p* < .0001; svPPA: *t*(92) = 4.61, *p* < .0001) and from baseline to 6 months (lvPPA-1: *t*(87) = 9.51, *p* < .0001; lvPPA-2: *t*(91) = 6.94, *p* < .0001; PCA: *t*(63) = 4.64, *p* < .0001; svPPA: *t*(92) = 9.36, *p* < .0001) (Fig. 3). Mean thickness reductions from baseline to 3 months ranged from 0.02 to 0.032 mm, representing 1.1–1.7% decreases from baseline, whereas thickness reductions from baseline to 6 months ranged from 0.039 to 0.05, representing 2–2.9% decreases from baseline. The lvPPA-1, PCA, and svPPA participants additionally showed longitudinal atrophy in core regions from 3 to 6 months follow-up (lvPPA-1: *t*(87) = 4.05, *p* < .0001; PCA: *t*(63) = 4.64, *p* < .0001; svPPA: *t*(92) = 4.88, *p* < .0001. Mean thickness reductions during this interval in these participants were ~0.02 mm, representing 1.2% decreases from 3 months follow-up. We found evidence of longitudinal atrophy in control regions much less systematically, only from baseline to 3 months in lvPPA-2 (*t*(91) = 3.07, *p* ≤ .003), with a 0.033 mm/1.5% decrease from baseline, and from 3 to 6 months in PCA (*t*(63) = 3.90, *p* < .0001), with a 0.037 mm/1.6% decrease from 3 months follow-up.

Next, we tested the critical interaction between the core and control ROIs to ask whether atrophy was preferential to the cortical regions that are phenotypically vulnerable. In all four participants, we found a significant interaction between Timepoint and ROI (Table 3). In each participant, we compared the magnitude of longitudinal atrophy (i.e., a decrease in thickness) from a given timepoint to another between core and control regions. In three participants (lvPPA-1, PCA, and svPPA), this interaction effect was driven by core regions exhibiting longitudinal atrophy of a greater magnitude from baseline to 3 months (lvPPA-1: Δ = 0.021 mm, 95% confidence interval [CI] = 0.01–0.032, *t*(88) = 3.86, *p* < .0001; PCA: Δ = 0.041 mm, 95% CI = 0.022–0.06, *t*(64) = 4.27, *p* < .0001; svPPA: Δ = 0.02 mm, 95% CI = 0.009–0.031, *t*(93) = 3.52, *p* ≤ .001) and from baseline to 6 months (lvPPA-1: Δ = 0.045 mm, 95% CI = 0.034–0.056, *t*(88) = 8.15, *p* < .0001; PCA: Δ = 0.027 mm, 95% CI = 0.007–0.046, *t*(64) = 2.67, *p* ≤ .01; svPPA: Δ = 0.031 mm, 95% CI = 0.02–0.043, *t*(93) = 5.55, *p* < .0001) compared with control regions. In the lvPPA-1 participant, core regions additionally showed greater longitudinal atrophy than control regions from 3 to 6 months: Δ = 0.024 mm, 95% CI = 0.013–0.035, *t*(88) = 4.26, *p* < .0001. In the lvPPA-2 participant, core regions showed greater longitudinal atrophy than control regions from 3 to 6 months (Δ = 0.024 mm, 95% CI = 0.007–0.042, *t*(92) = 2.72, *p* ≤ .008), which was partially driven by a non-significant negative difference observed with the latter (Δ = -0.014 mm, 95% CI = -0.035 to 0.007, *t*(91) = -1.36, *p* ≤ .18).

### 3.2 Vertex-wise analysis reveals the possible spatiotemporal trajectory of short-interval longitudinal cortical atrophy

**Fig. 4.**
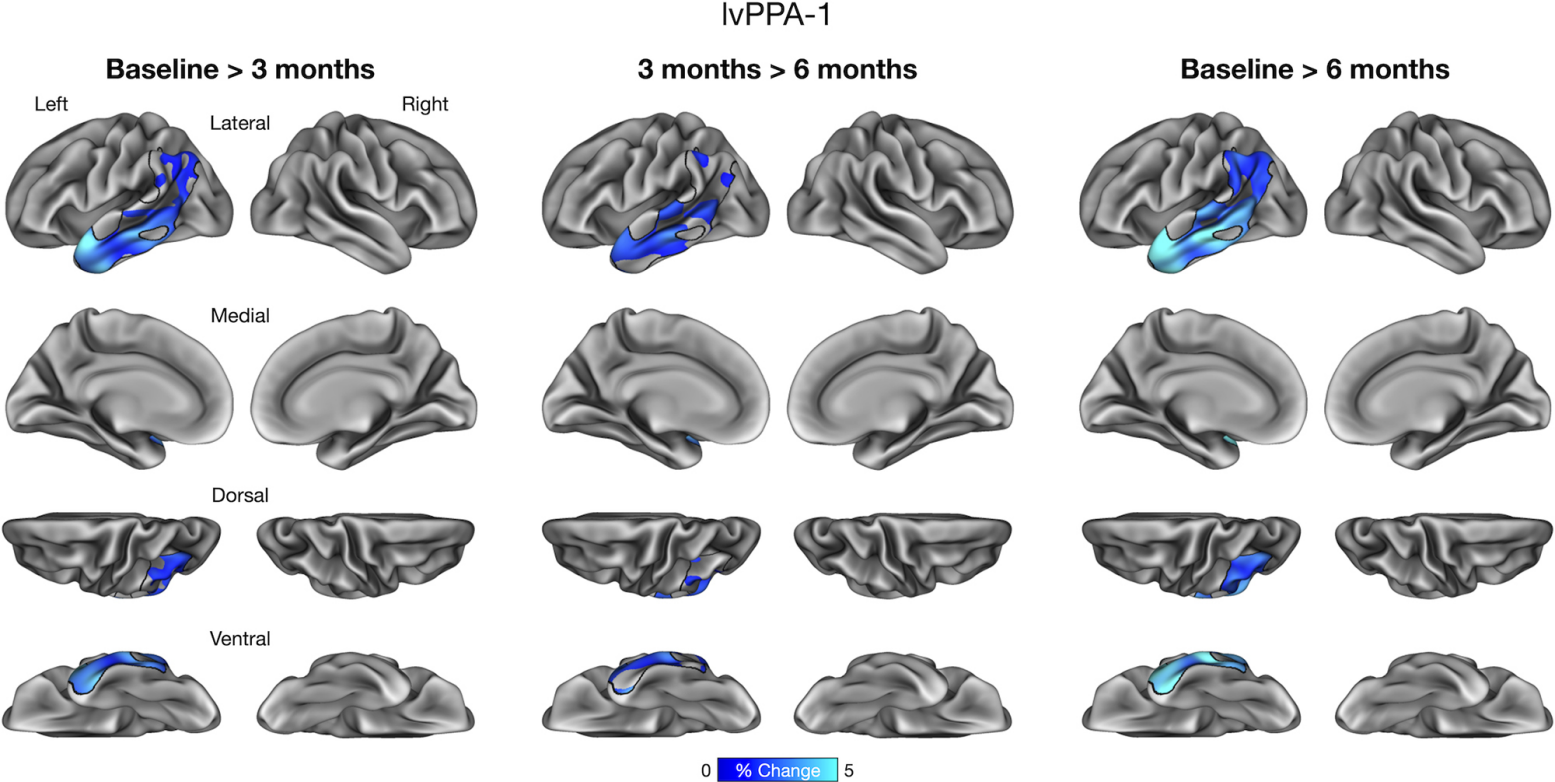
Vertex-wise analysis of longitudinal cortical atrophy within core atrophy ROIs in a patient with logopenic variant Primary Progressive Aphasia (lvPPA-1).

**Fig. 5.**
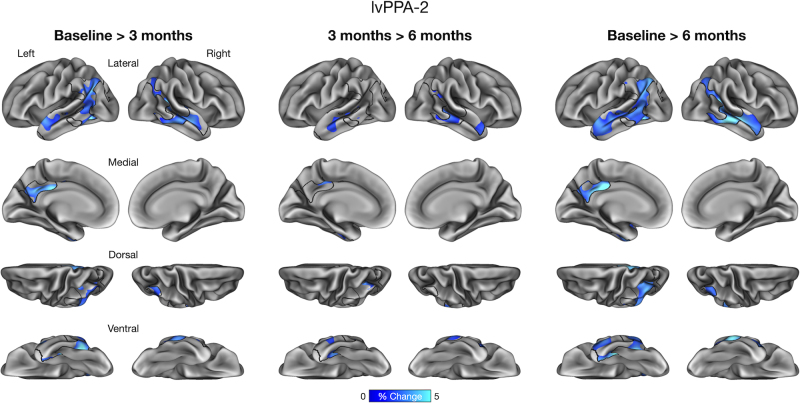
Vertex-wise analysis of longitudinal cortical atrophy within core atrophy ROIs in a patient with logopenic variant Primary Progressive Aphasia (lvPPA-2).

**Fig. 6.**
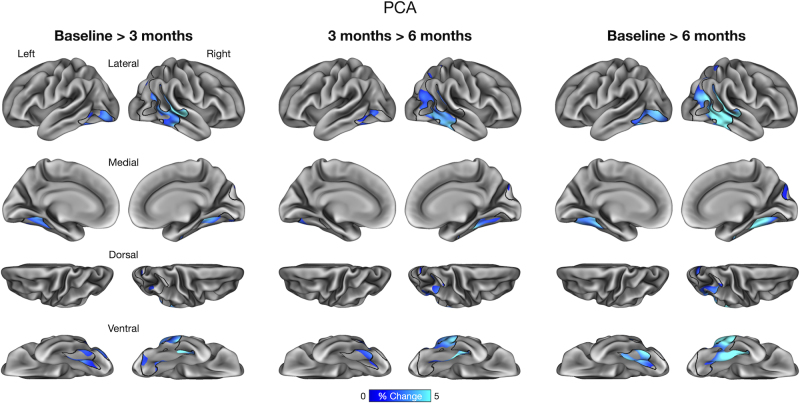
Vertex-wise analysis of longitudinal cortical atrophy within core atrophy ROIs in a patient with Posterior Cortical Atrophy (PCA).

**Fig. 7.**
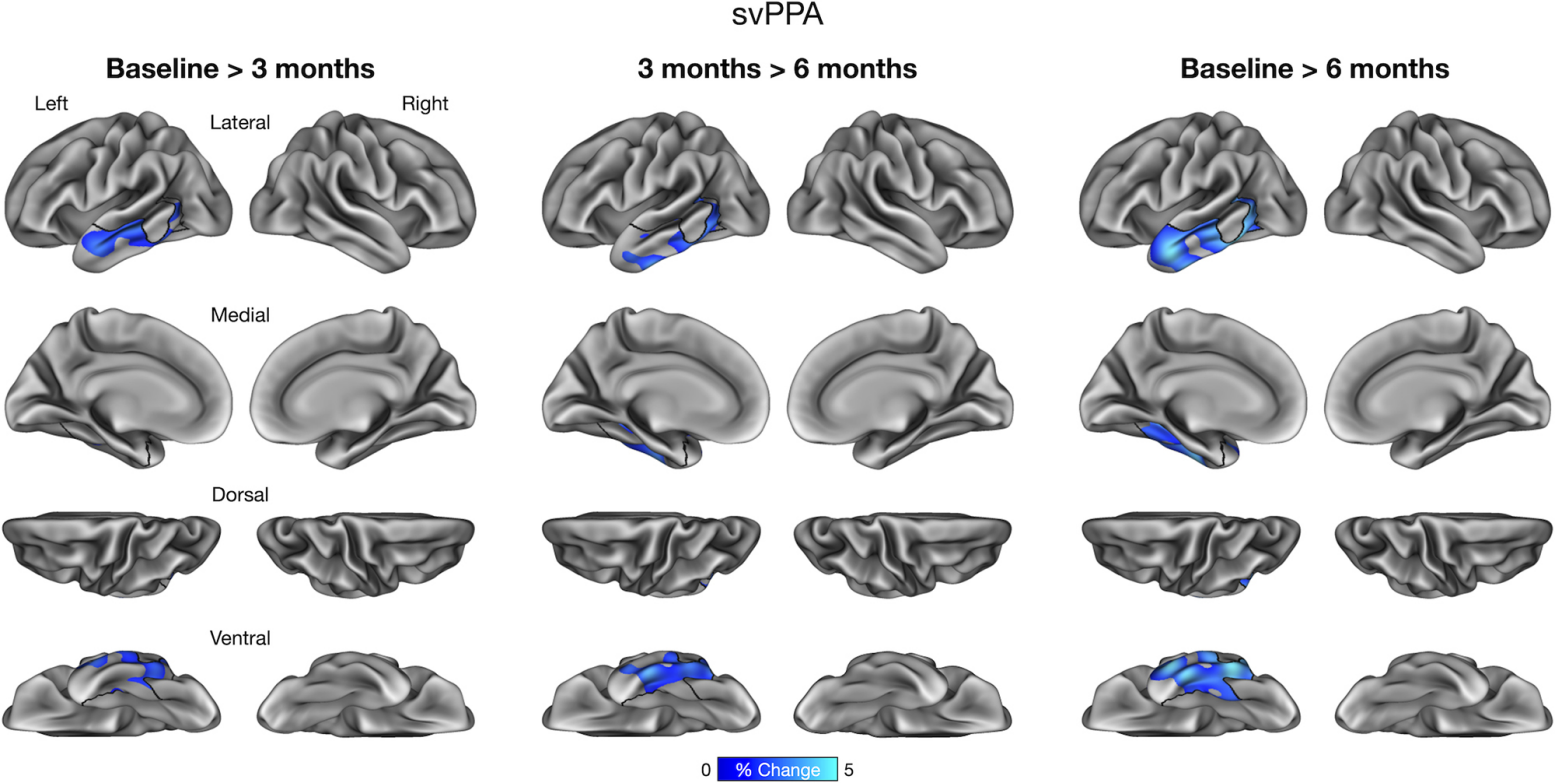
Vertex-wise analysis of longitudinal cortical atrophy within core atrophy ROIs in a patient with semantic variant Primary Progressive Aphasia (svPPA).

We performed an exploratory vertex-wise analysis of longitudinal cortical atrophy within the boundaries of core atrophy regions in each participant. This analysis identified prominent longitudinal atrophy within each participant’s phenotypically vulnerable cortical regions consistent with ROI-based results reported above. In lvPPA-1, we found longitudinal atrophy from baseline to 3 months prominently in the anterior lateral temporal lobe and inferior parietal lobule, which continued from 3 to 6 months. By 6 months, longitudinal atrophy was detectable throughout core regions (Fig. 4). In lvPPA-2, we identified longitudinal atrophy primarily in the lateral temporal cortex, inferior parietal lobule, and precuneus/posterior cingulate cortex from baseline to 3 months, with modest changes observed from 3 to 6 months. By 6 months, longitudinal atrophy became more spatially extensive within the ROI similarly to lvPPA-1 (Fig 5). In PCA, we found longitudinal atrophy from baseline to 3 months localized to ventral and lateral temporo-occipital areas, which continued from 3 to 6 months. Longitudinal atrophy was slightly more pronounced in the right middle and inferior temporal gyri over 6 months, whereas the dorsal aspects of the ROI (e.g., right superior parietal lobule) exhibited minimal changes over this interval (Fig. 6). Finally, in svPPA, we identified longitudinal atrophy from baseline to 3 months localized to the left anterior lateral temporal cortex, with thickness changes in other areas of the lateral temporal cortex from 3 to 6 months. By 6 months, longitudinal atrophy was greater in its extent, covering most areas of the left-lateralized ROI mask (Fig. 7).

### 3.3 Data pooling improves the precision in estimating short-interval longitudinal atrophy

**Fig. 8.**
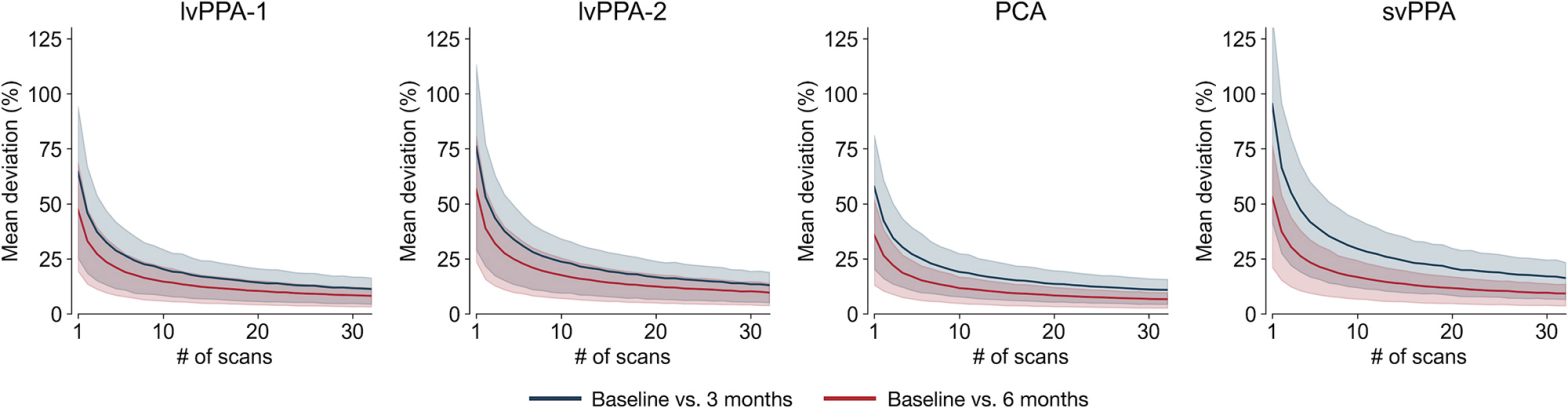
Data pooling improves the precision in estimating short-interval longitudinal atrophy. Each plot represents the percent deviation (*y*-axis) in longitudinal atrophy estimates derived from different numbers of fast structural MRI scans (*x*-axis). Shaded bands represent the interquartile ranges.

Finally, we estimated the magnitude and precision of longitudinal cortical atrophy within each participant using varying numbers of fast structural MRI scans from each timepoint. As expected, the magnitude of percent deviation in longitudinal atrophy estimation (relative to the reference value obtained from 32 scans per timepoint) progressively declined as a function of the number of scans across which thickness estimates were pooled at each timepoint (Fig. 8). Using this metric, we next estimated the number of scans per timepoint where improvement in estimation precision due to data pooling was minimal. This number ranged from 11 to 14, with the corresponding percent deviation ranging from 11% to 28% (Table 5).

**Table 5.** Number of scans to estimate longitudinal atrophy with minimal improvement in precision (<5%).

	Time interval	
Participant ID	BL to 3 months	BL to 6 months
lvPPA-1	14 (16.96%)	13 (12.93%)
lvPPA-2	13 (20.83%)	12 (15.95%)
PCA	13 (16.86%)	11 (11.37%)
svPPA	11 (28.24%)	12 (15.19%)

Notes: Values represent the number of fast structural MRI scans where improvement in estimation precision was no more than 5% of the immediately preceding deviation value for at least two consecutive steps. Values in parentheses represent the corresponding percent deviation.

## 4 Discussion

The goal of this study was to evaluate the utility of dense sampling of structural MRI data at each timepoint—*cluster scanning*—in the measurement of longitudinal cortical atrophy over short time intervals by means of increasing brain morphometric precision. In each individual patient with AD or FTLD, we found that phenotypically vulnerable cortical (“core atrophy”) regions exhibited longitudinal atrophy that was detectable over 3- and 6-month intervals. Moreover, the magnitude of longitudinal atrophy in core regions was statistically greater than the magnitude of atrophy observed in control regions over 6 months and at least one of the 3-month intervals in three of four participants; this finding additionally demonstrates the utility of cluster scanning for detecting selective longitudinal atrophy in vulnerable regions over these intervals. An exploratory vertex-wise analysis revealed subregional specificity in the localization of longitudinal atrophy within phenotypically vulnerable regions, the extent of which was variable across individual participants. Taken together, these findings provide proof-of-concept evidence demonstrating the utility of MRI cluster scanning for the detection of progressive neurodegeneration over intervals that are half or even less than half of those typically used in large, multicenter natural history and biomarker studies such as ADNI, DIAN, ALLFTD, LEADS, PPMI, and many others.

The current results support that cortical regions already exhibiting atrophy in the early symptomatic stages of patients with neurodegenerative disease continue to do so over the following 6-month period. This is consistent with prior work identifying partial spatial overlap in the localization of baseline atrophy and longitudinal atrophy among individuals with lvPPA, PCA, and svPPA who were mildly impaired at baseline (Kumfor et al., 2016; Rohrer et al., 2013; Sintini et al., 2020). In the lvPPA-2 participant, we found that the rate of longitudinal atrophy within the phenotypically vulnerable regions was no longer statistically significant in the second 3-month interval (from 3 to 6 months follow-up). It is possible that some portions within these regions had become saturated with neurodegenerative changes and the leading edge of atrophy shifted, thus reducing the overall magnitude of cortical atrophy detectable during this period. Our exploratory vertex-wise analysis provided support for this possibility, where significant atrophy was identified in vertices spatially distinct from those exhibiting atrophy during the initial 3-month interval in the lvPPA-2 participant. While a provisional observation, this post-hoc exploration suggests it may be possible to refine procedures in the future to track the dynamic evolution of neurodegeneration with dense, repeat sampling procedures.

In addition, our findings suggest that the time-dependent changes in cortical thickness do not represent a global phenomenon, as indicated by longitudinal atrophy of greater magnitudes in core regions than in control regions observed in the majority of the participants. We found comparable magnitudes of thickness change between core and control regions in the lvPPA-2 participant. This participant’s control ROI was substantially smaller than their core ROI, indicating that more extensive vertices within the broader primary sensorimotor cortex showed evidence of baseline atrophy. The resulting smaller control ROI size might have led to higher measurement variability across the repeated acquisitions at each timepoint. Although our modeling approach allowed separate residual variances for each ROI, reduced measurement precision in the smaller control region might have contributed to reduced statistical power for detecting differential atrophy patterns. Future work should employ alternative approaches to defining control regions, such as completely data-driven identification of minimally atrophic areas while equating ROI sizes.

Finally, we found in each participant that pooling thickness estimates from greater numbers of scans per timepoint leads to progressive improvement in estimation precision. This is consistent with the findings from our prior cluster scanning studies identifying similar benefits from data pooling in boosting the precision of estimating hippocampal volume and other regional morphometric features in aging and neurodegenerative dementias (Elliott et al., 2024, 2025). Although low-resolution structural MRI scans employed in this study can be acquired more quickly than conventional 1-mm isotropic MRI scans, collecting 32 scans per timepoint over 2 days would significantly increase participant burden and study cost. Such a design would also be impractical in typical neuroimaging studies. In this context, cluster scanning using extremely rapid sequences will help boost morphometric precision necessary for the detection of short-interval atrophy while maintaining a reasonable scan duration. We previously leveraged extremely rapid MRI sequences with 1.0 mm isotropic resolution, including a compressed-sensing acquisition with six-fold acceleration (CS; acquisition time = 1’12”) (Mussard et al., 2020) and an acquisition with wave-controlled aliasing in parallel imaging and 3 × 3 acceleration (WAVE × 9; acquisition time = 1’9”) (Polak et al., 2018). Despite the five-fold reduction in acquisition time, we found that a single scan collected with these sequences produces morphometric estimates with high test-retest reliability, high convergent validity, and measurement error rate comparable to those obtained from a standard ADNI MRPAGE scan (acquisition time = 5’12”) (Elliott et al., 2023).

In addition, we recently demonstrated that longitudinal atrophy can be robustly detected in patients with cognitive impairment and in some cognitively unimpaired older adults over 1 year using clusters of eight CS MRI scans (just under 10 minutes) (Elliott et al., 2025). In the current study, we found that collecting 11–14 scans per timepoint might represent a reasonable trade-off between the number of scans/scan duration and morphometric precision when estimating longitudinal atrophy over 3–6 months. When acquired via CS MRI sequences, these scans would translate to approximately 13–17 minutes of imaging per timepoint. This might be tolerable and even possible to achieve in a single scanning session in cognitively impaired individuals with AD or FTLD; the short acquisition duration per scan would also help reduce the impact of imaging artifacts due to head motion, thus minimizing the chance of potential rescans and subsequent data removal. It is also likely that, in the near future, scans utilizing deep learning reconstructions will further accelerate acquisition times and image quality.

Beyond the proof-of-concept evidence that supports further investigation of the cluster scanning approach, several questions remain. First, future work should investigate the pattern of short-interval longitudinal atrophy in more diverse samples, including individuals with different clinical phenotypes of AD/FTLD (e.g., early-onset AD, behavioral variant FTD) and disease stages, where the magnitude and spatial extent of cortical atrophy may increase at variable rates (Cho et al., 2013; E. Gordon et al., 2021; Staffaroni et al., 2020). Studying such individuals over a large number of short intervals (e.g., every 3–6 months for 2–4 years) would help better understand the heterogeneity in disease progression over finer temporal scales in the context of differences in the topography of neuroanatomical abnormalities and in the molecular drivers of neurodegeneration. In addition, it will be important to examine age-matched, cognitively unimpaired participants in which the rate of longitudinal atrophy over such intervals is usually much smaller in magnitude compared with that detectable in neurodegenerative patients. Establishing this reference based on control data, as we are beginning to do (Elliott et al., 2025), would facilitate the interpretation of the magnitude of longitudinal atrophy in patients and would also be useful in accounting for age dependency in the observed effects. It will also be important in future longitudinal studies to acquire standard T1w structural MRI scans (e.g., ADNI MPRAGE) so that estimates of longitudinal atrophy can be directly compared against those obtained through cluster scanning (Elliott et al., 2025).

Second, future studies should also perform external validation of short-interval longitudinal atrophy as measured by cluster scanning against other biomarkers that are used in natural history studies and clinical trials, including amyloid PET, tau PET, ^18^F-fluorodeoxyglucose PET, and plasma and cerebrospinal fluid measures. Clarifying the relationships among these biomarkers would be essential in evaluating and predicting the trajectory of neurodegenerative change in individual patients. For instance, it would be beneficial to understand how the level of cerebral tau accumulation or neurofilament light predicts the rate of cortical atrophy over the next several months. This information may be useful in estimating the rate of downstream cognitive and functional decline, particularly among individuals following an aggressive clinical course.

Third, in this study we focused on a set of cortical regions that had already been showing evidence of atrophy prior to baseline in each participant. We hypothesized that these regions would continue to show progressive atrophy over time given that our AD/FTLD participants were in the early clinical stages of their disease. This was confirmed in our preliminary data analysis, where we found longitudinal thickness changes of a greater magnitude overall in the core atrophy ROIs compared with the “penumbra” ROIs (-2 ≤ *W* < -1). Our vertex-wise analysis additionally revealed evidence showing progressive atrophy within the boundaries of the core ROIs over the 6-month study period. Nonetheless, future investigations would benefit from examining a broader set of cortical regions beyond those that are phenotypically vulnerable, to more comprehensively map the trajectory of longitudinal atrophy within each participant.

Fourth, given that the magnitude of longitudinal change in cortical thickness is small over 3- and 6-month intervals, it would be important for future studies to evaluate potential effects of partial volume fractions that exist between the pial and white surfaces on thickness estimation. Methods that address this issue are emerging in the literature (e.g., Joshi et al., 2025), which would allow systematic quantitative comparisons of atrophy rates across approaches (Clarkson et al., 2011; Tustison et al., 2014).

Finally, cluster scanning could be helpful in refining our understanding of the effects of anti-amyloid monoclonal antibodies on regional brain volume, recognizing the counterintuitive findings to date regarding reductions in brain volume with amyloid-lowering therapies (Belder et al., 2024). We would hope that a comparison of short-interval cluster scanning-based morphometric measurements in cortical areas of high versus low baseline amyloid burden would clarify whether MRI volumetric reductions are specifically localized to areas where amyloid is being removed and not other areas, supporting the concept of pseudo-atrophy.

In sum, the present findings strongly suggest the feasibility of robustly detecting longitudinal cortical atrophy over short intervals within individual patients with suspected AD or FTLD via MRI cluster scanning. These findings have implications for the development of small, early phase, or even personalized (“*N*-of-1”) clinical trials examining disease-modifying effects of therapeutic interventions. Specifically, using cluster scanning, it is conceivable to estimate the natural progression of neurodegeneration in an individual patient across three timepoints over a 6-month run-in phase, followed by an intervention phase that involves MRI assessments every 3 months for the duration of the trial to precisely identify the timing of its effect on slowing the rate of neurodegeneration. If successful, such a design could dramatically alter the landscape of clinical trials, while also potentially supporting the evaluation of crossover to different treatments (e.g., as in platform trials) to offer patients the maximal opportunity for benefit and to gain the most knowledge from each individual.

## Data Availability

MRI data used in this manuscript may be shared (anonymized) at the request of any qualified investigator for purposes of replicating procedures and results. Data analysis code is available at https://github.com/yutakatsumi/long-cluster-scan.
